# Social and monetary reward processing in autism spectrum disorders

**DOI:** 10.1186/2040-2392-3-7

**Published:** 2012-09-26

**Authors:** Sonja Delmonte, Joshua H Balsters, Jane McGrath, Jacqueline Fitzgerald, Sean Brennan, Andrew J Fagan, Louise Gallagher

**Affiliations:** 1 Department of Psychiatry, Trinity College Dublin, Dublin, 2, Ireland; 2 Trinity College Institute of Neuroscience, Trinity College Dublin, Dublin, 2, Ireland; 3 Centre for Advanced Medical Imaging (CAMI), St. James’s Hospital / Trinity College, Dublin, 8, Ireland

**Keywords:** Autism, Reward, Social motivation, Striatum, Functional magnetic resonance imaging, fMRI

## Abstract

**Background:**

Social motivation theory suggests that deficits in social reward processing underlie social impairments in autism spectrum disorders (ASD). However, the extent to which abnormalities in reward processing generalize to other classes of stimuli remains unresolved. The aim of the current study was to examine if reward processing abnormalities in ASD are specific to social stimuli or can be generalized to other classes of reward. Additionally, we sought to examine the results in the light of behavioral impairments in ASD.

**Methods:**

Participants performed adapted versions of the social and monetary incentive delay tasks. Data from 21 unmedicated right-handed male participants with ASD and 21 age- and IQ-matched controls were analyzed using a factorial design to examine the blood-oxygen-level-dependent (BOLD) response during the anticipation and receipt of both reward types.

**Results:**

Behaviorally, the ASD group showed less of a reduction in reaction time (RT) for rewarded compared to unrewarded trials than the control group. In terms of the fMRI results, there were no significant group differences in reward circuitry during reward anticipation. During the receipt of rewards, there was a significant interaction between group and reward type in the left dorsal striatum (DS). The ASD group showed reduced activity in the DS compared to controls for social rewards but not monetary rewards and decreased activation for social rewards compared to monetary rewards. Controls showed no significant difference between the two reward types. Increased activation in the DS during social reward processing was associated with faster response times for rewarded trials, compared to unrewarded trials, in both groups. This is in line with behavioral results indicating that the ASD group showed less of a reduction in RT for rewarded compared to unrewarded trials. Additionally, de-activation to social rewards was associated with increased repetitive behavior in ASD.

**Conclusions:**

In line with social motivation theory, the ASD group showed reduced activation, compared to controls, during the receipt of social rewards in the DS. Groups did not differ significantly during the processing of monetary rewards. BOLD activation in the DS, during social reward processing, was associated with behavioral impairments in ASD.

## Background

Autism spectrum disorders (ASD) are characterized by deficits in social communication and restricted interests and repetitive behaviors [1]. ‘Social motivation theory’ proposes that deficits in social interaction are due to a difficulty in forming reward representations of social stimuli, which results in reduced social attention and contributes to further difficulties in terms of social interaction and communication [2-4]. Restricted interests and repetitive behavior may, on the other hand, reflect hyper-responsive activity in reward circuits to certain classes of stimuli [5]. Therefore, studying the neural basis of reward processing in ASD provides a promising approach to understanding core deficits in ASD.

Reward processing involves a well defined, interconnected, network of cortical and subcortical regions including the orbitofrontal (OFC) and ventromedial prefrontal cortex (vmPFC), anterior cingulate cortex (ACC), striatum, amygdala, and the dopaminergic midbrain [6-9]. Neuroimaging techniques allow the dissociation of neural mechanisms involved in ‘wanting’ referring to the incentive motivation to seek the reward and ‘liking,’ referring to the hedonic value of the reward [10]. Anticipation (‘wanting’) of rewards is typically associated with activity in the ventral striatum (VS) whereas receipt (‘liking’) is associated with vmPFC activity [11]. The OFC is associated with coding stimulus reward value, the amygdala with tracking emotional salience of stimuli, and the ACC with conflict monitoring [7,8]. The striatum is critical to this circuit; the ventral striatum (VS) for the motivational control of action and dorsal striatum (DS) for integrating rewards with executive functions and action control [9,12].

Social reward processing involves a number of neural regions associated with primary (for example, food) and secondary (for example, monetary) rewards. Social reward paradigms have used attractive faces, positive feedback (for example, a smiling face), and more complex social situations such as acquiring a good reputation [13-15]. Beautiful faces activate foci in the VS and OFC [15] and anticipation of positive emotional expressions has been shown to activate the VS [13,16]. Common activation during the receipt of social and monetary rewards has been reported in the striatum [17] and social and monetary reward learning engage shared regions of vmPFC and striatum [18]. On the other hand, the amygdala has been associated with the receipt of social but not monetary rewards in one study [16] and the DS has been implicated in the receipt of complex social rewards [19-22], suggesting that some regions may be more involved in processing social rewards.

Previous studies in ASD suggest abnormalities in both social and monetary reward processing. Reduced activation in the VS has been reported during social but not monetary reward feedback in children with ASD [23] as well as during the anticipation of monetary but not social rewards [24,25]. Reduced VS and vmPFC activity has been reported during the receipt of monetary rewards [5] as well as increased activity in the ACC [24,26] and OFC [23]. Both increases and decreases in amygdala activation have been reported during social reward anticipation and receipt [24,25] and decreased amygdala activation has been recorded during the receipt of monetary rewards [25]. The results of these studies are clearly heterogeneous and suggest that deficits in reward processing in ASD may be non-specific extending to classes of stimuli beyond social rewards and involving a number of regions within reward circuitry.

In this study we used adapted versions of the monetary and social incentive delay tasks (MID and SID) [11,13,16,27] to examine reward processing among unmedicated participants with high functioning ASD. A factorial design was used to test two hypotheses: (1) that there is a general dysfunction in reward processing in ASD (main effect of group), characterized by abnormal BOLD responses during the anticipation and/or receipt of both monetary and social rewards, as suggested by the results of previous fMRI studies [5,23-26]; and (2) that there is a specific deficit in social reward processing (group by reward type interaction), characterized by reduced activation during the anticipation and/or receipt of social rewards, in line with social motivation theory [2]. Based on anatomical regions highlighted by social motivation theory [28], previous studies of social and monetary reward processing [11,13,16,17] and studies of reward deficits in ASD [5,23-26] we predicted that group differences in reward processing would be localized to the vmPFC, OFC, ACC, amygdala, and/or striatum. In addition, we sought to explore the relationship between abnormal BOLD responses to rewards and behavioral impairments in ASD.

## Methods

### Participants

Twenty-one right-handed Caucasian ASD (mean age, 17.64 (3.45) years; age range, 13.58 to 25.91 years) and 21 right-handed Caucasian control participants (mean age, 17.00 ± 3.37 years; age range, 12.04 to 25.66 years) were included in the analyses. ASD participants were recruited through an associated genetics research program, clinical services, schools, and advocacy groups. Controls were recruited through schools, the university, and volunteer websites. Ethical approval was obtained from St. James’s Hospital/AMNCH (ref: 2010/09/07) and the Linn Dara CAMHS Ethics Committees (ref: 2010/12/07). Written informed consents/assents were obtained from all participants and their parents (where under 18 years of age).

Exclusion criteria included a Full Scale IQ (FSIQ) <70, known psychiatric, neurological, or genetic disorders, a history of a loss of consciousness for >5 min and those currently taking psychoactive medication. Four subjects in the ASD group had a secondary diagnosis of Attention Deficit Disorder (ADD) or Attention Deficit Hyperactivity Disorder (ADHD). Controls were excluded if they had a first-degree relative with ASD or scored >50 on the Social Responsiveness Scale (SRS) [29] or >10 on the Social Communication Questionnaire (SCQ) [30]. The adult prepublication version of the SRS was used with permission in cases 18 years or older [31]. All participants had normal, or corrected to normal, vision.

### Diagnostic assessments and cognitive measures

ASD diagnosis was confirmed using the Autism Diagnostic Observation Schedule (ADOS) [32] and the Autism Diagnostic Interview Revised (ADI-R) [33]. All ASD participants met criteria for autism on the ADI-R. Twelve participants met criteria for autism and nine met criteria for ASD on the ADOS. Clinical consensus diagnosis was established using DSM-IVTR criteria and expert clinician (LG).

FSIQ was measured using the four-subtest version of the Wechsler Abbreviated Scale of Intelligence (WASI; [34]) or the Wechsler Intelligence scale for Children-Fourth Edition (WISC-IV; [35]). Performance IQ (PIQ) score was based on the Matrix Reasoning and Block Design subtests and Verbal IQ (VIQ) score on the Vocabulary and Similarities subtests.

### Functional MRI tasks

**Figure 1:**
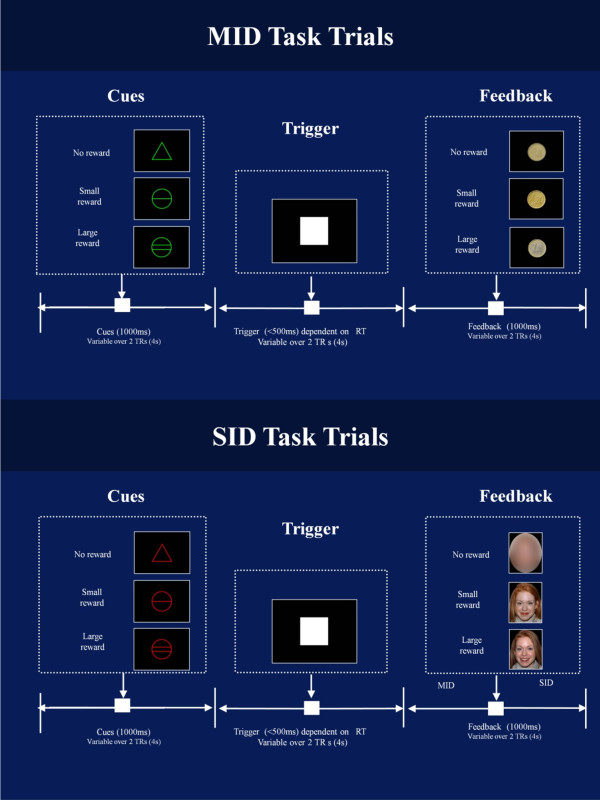
** MID task trials (top panel) and SID task trials (bottom panel).** Each trial was divided into three 4-s periods; cues occurred in the first period (0 to 4 s), triggers in the second (4 to 8 s) and feedback in the third (8 to 12 s). Cues, triggers, and feedback occurred pseudo-randomly within these 4-s periods so that activity time-locked to each event type was uncontaminated by preceding or proceeding trial elements.

Figure 1 illustrates the adapted versions of the MID [27] and the SID [13]. In both tasks participants had to respond as quickly as possible to a trigger (white square) while it remained on screen. The amount of time the participant had to respond to the trigger depended on the number of correct or incorrect prior responses (see below). Trigger cues were preceded by an instruction cue signaling the level of potential reward. For ‘reward’ trials a circle denoted that participants would be rewarded if they responded quickly enough (*n* per task = 60) while for ‘no reward’ trials a triangle denoted that the participant would not receive a reward, regardless of whether or not they responded quickly enough to the trigger (*n* = 30). Reward magnitude varied on two levels indicated by the number of horizontal lines on a cue stimulus. In the MID the levels of monetary reward were €0.20 (*n* = 30, preceded by a cue depicting a circle with one horizontal line) and €1.00 (*n* = 30, preceded by a cue showing a circle with two horizontal lines). Success was acknowledged by showing a picture of a coin with the money earned on that trial. In the case of a ‘no reward’ trial, or when participants did not respond to the trigger quickly enough, they were shown a coin stimulus of the same size and luminance but with no features. SID instruction cues were identical to MID instruction cue except in color. Feedback was a female face from the NimStim set of Facial Expressions [36] with a happy facial expression at two levels of intensity (small smile and larger smile), as used in previous studies of social reward [16]. This face stimulus was presented as it was rated as the most pleasant and attractive of the Caucasian faces in the NimStim set by a sample of 20 male participants (see supplementary material) and was used previously in a study of social reward in children [37]. Unlike the original SID task, which used 22 different faces, a single female face was used to remove novelty as a confounding difference between tasks. Two levels of social reward, rather than three (as in the original SID task), were used to reduce task duration. The ‘no reward’ facial stimulus was the same face graphically dysmorphed, with facial features eliminated but size and luminance retained. 

Each task consisted of 90 trials (two 45 trial runs (TR) each lasting 9 min) presented in a counterbalanced order across participants. Each trial lasted 12 s (six TRs). A variable delay was introduced between the instruction cue and trigger (1,492 to 6,848 ms), and trigger and feedback (1,417 to 6,569 ms) to ensure that BOLD activity time-locked to the instruction cue was specific to reward anticipation and uncontaminated by the subsequent response or feedback. Similarly, activity at the time of feedback was specific to reward receipt and uncontaminated by reward anticipation or motor responses [38,39]. This variable delay was achieved by randomly varying the onset time of instruction cues, triggers and feedback across the first two TRs (0 to 4 s), second two TRs (4 to 8 s) and last two TRs (8 to 12 s), respectively, from trial to trial. Cues and feedback were each presented for 1,000 ms. As in previous MID studies, the duration of the trigger was adjusted to maintain an accuracy rate for approximately two-thirds of trials. Response periods were reduced by 30 ms after each correct response, and increased by 90 ms when participants failed to respond within the given time frame. Manipulations of the response period were separate for each reward level given that RTs are known to be faster for higher levels of reward [11,27,40]. An upper limit was imposed, such that trigger duration could not exceed more than 500 ms.

Participants maintained focus on the cross hair in the center of the screen throughout the fMRI sessions. They were instructed to respond quickly to the trigger using a button in their right hand. For the MID they were told that they could ‘win’ real money up to a value of €30. All subjects were given €25 at the end of the experiment, regardless of their performance. For the SID they were informed that success would be acknowledged by a smiling face on the screen. Practice versions of each task (consisting of 30 trials) were performed to familiarize participants with the experiments prior to scanning.

### fMRI data acquisition

MRI data were collected on a Philips 3 T Achieva MRI Scanner at the Centre for Advanced Medical Imaging (CAMI), St. James’s Hospital, Dublin. A high-resolution 3D T_1_-weighted MPRAGE image was acquired for each participant (FOV, 256×256×160 mm^3^; TR, 8.5 ms; TE, 3.9 ms; total acquisition time, 7.3 mins; voxel size, 1×1×1 mm^3^). Two hundred and eighty functional images were acquired for each run using a T_2_* weighted gradient echo sequence to visualize changes in the BOLD signal (TR, 2,000 ms; TE, 28 ms; flip angle, 90°; FOV, 256×256 mm^2^; voxel size, 3×3×3.5 mm^3^; slice gap, 0.35 mm; 38 slices; slice order scan order: ascending; total acquisition time, 9.3 min). Presentation® software (Version 14.4, http://www.neurobs.com) was used for stimulus presentation. Subjects lay supine and stimuli were projected onto a screen behind the subject and viewed in a mirror above the subject’s face.

### Statistical analysis of behavioral data

Behavioral data were analyzed using SPSSv16. Two sample t-tests were used to examine group differences in age, IQ measures, SRS, and SCQ scores. Mixed model (between/within subjects) ANOVAs were used to examine accuracy and reaction time (RT) data. Pearson’s correlations were conducted to examine the relationship between the BOLD response and SRS score and RT. Correlations between BOLD response and ADOS/ADI scores were calculated using Spearman’s rho, as ADOS/ADI scores are ranked/ordinal. Correlations were corrected for multiple comparisons using Bonferroni correction.

### fMRI data analysis

fMRI analysis was carried out in SPM8 ( http://www.fil.ion.ucl.ac.uk/spm) in Matlab 2009a (MathWorks Inc., UK). Before preprocessing, the origin was set to the anterior commisure for both T_1_-weighted and EPI images. Slice-timing correction was then applied to the data, given the recent evidence that this approach is superior to flexible modeling strategies in correcting for differences in image acquisition time between slices [41]. The images were then realigned to correct for motion artefacts and co-registered to the skull stripped T_1_-weighted image. Subjects (ASD, *n* = 6; controls, *n* = 4; additional to the 21 cases/controls presented here) were excluded for excessive head motion during scanning (that is, movements >3 mm). Normalization to standard stereotaxic space (Montreal Neurological Institute; MNI) was performed using the ICBM EPI template and the unified segmentation approach [42]. The data were then re-sliced to a voxel size of 2×2×2 mm^3^. Finally, the images were smoothed using a 5-mm full-width-half-maximum (FWHM) Gaussian kernel to conform to assumptions of statistical inference using Gaussian Random Field Theory [43,44].

Nine event types were modeled at the first level for each task: anticipation/cue (‘no reward’, ‘small reward’, ‘large reward’, ‘error’), feedback (‘no reward’, ‘small reward’, ‘large reward’, ‘error’), and ‘trigger’. ‘Cue error’ and ‘feedback error’ comprised reward trials on which participants failed to respond within the given time frame. Nine regressors were created by convolving a delta function of event onset times for each event with the canonical hemodynamic response function (HRF). Given that slice time correction was used, micro-time onset was set to the middle temporal slice. Covariates of no interest included the six head motion parameters.

Following first level analysis contrast files were created to examine differences in BOLD response between ‘no reward’ and ‘reward’ (small and large combined) for both anticipation and feedback. The two levels of reward were combined as behavioral results indicated differences between ‘no reward’ and ‘reward’ rather than between the two reward levels. Second level random effects group analyses were used to examine the BOLD response to reward anticipation and feedback. Two two-by-two mixed model ANOVAs (between subjects factor: group; within subjects factor: reward type) were run, to examine main effects and interactions, one for reward anticipation and one for reward feedback. These were followed up using independent and paired sample t-tests. Whole brain analyses were thresholded at *P* <0.001 uncorrected (10 contiguous voxels). Finally, age and FSIQ were added as covariates to control for possible effects of these factors.

Key anatomical regions within the reward system (striatum, amygdala, vmPFC, OFC, and ACC) were defined *a priori* for small volume correction to correct for multiple comparisons at the family wise error rate (FWE; *P* <0.05). Masks for each of these regions were generated in FSL ( http://www.fmrib.ox.ac.uk/fsl/) using the Harvard Oxford cortical and subcortical atlases ( http://www.cma.mgh.harvard.edu/). The caudate nucleus, putamen, and nucleus accumbens were combined into a striatal mask (one for each hemisphere) using the image calculator in SPM8. All masks were thresholded at >20% probability. Percent signal change in significant activations was calculated using the Anatomy Toolbox [45] in SPM8.

## Results

Groups did not differ in terms of age, FSIQ, VIQ, or PIQ. There was a significant difference between groups on the SRS and the SCQ (see Table 1).

**Table 1:** **Mean scores for age, IQ, and scales ofsocial functioning**

	**Autism (*n*** **= 21)**	**Controls (*n*** **= 21)**	***P***
Age (years)	17.64 (3.45)	17.00 (3.37)	0.545
WASI			
Full Scale IQ	109.38 (15.94)	110.00 (12.53)	0.889
Verbal IQ	108.67 (15.23)	108.86 (14.14)	0.967
Performance IQ	107.48 (15.47)	109.33 (11.37)	0.660
Social Responsiveness Scale (SRS)	95.95 (27.22)	13.95 (11.40)	<0.001^a^
Social Communication Questionnaire (SCQ)	21.88 (6.37)	2.79 (2.97)	<0.001^a^

Standard deviations are shown in parenthesis.

^a^Significant group difference.

### Reaction time

**Figure 2:**
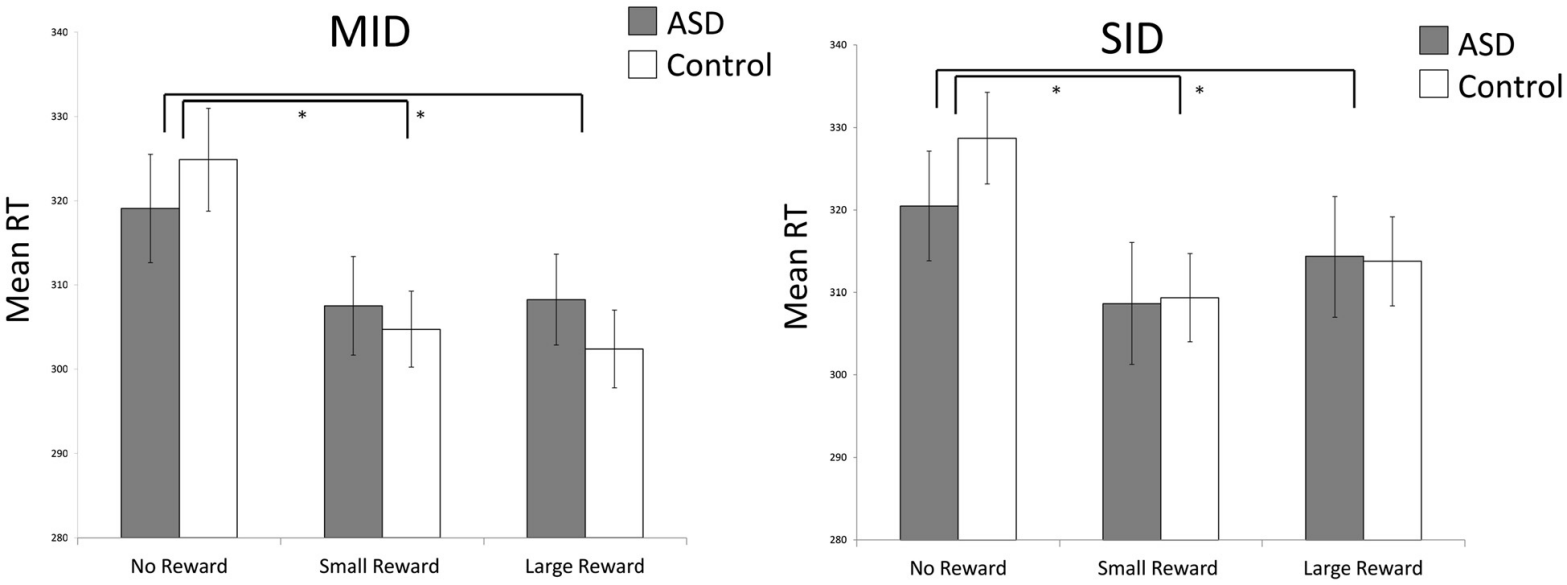
** Reaction time (RT) (ms) for MID and SID tasks.** RT is shown in gray for the ASD group and in white for the control group. Standard error of the mean is displayed and significant differences in RT between levels of reward magnitude are marked with an asterisk.

Reaction time values are shown in Figure 2. A mixed model two-by-two-by-three ANOVA (between-subjects factor: group; within subjects factors: reward type and reward magnitude) revealed a significant effect of reward magnitude (*F* (1.61, 64.33) = 47.49, *P* <0.0001; faster responses to ‘reward’ compared to ‘no reward’) and a significant interaction between group and reward magnitude, (*F* (1.61, 64.33) = 4.70, *P* = 0.018). Pair-wise comparisons to examine the main effect of reward magnitude indicated a significant decrease in RT between ‘no reward’ and ‘small reward’ levels (t(41) = 8.660, *P* <0.0001) as well as ‘no reward’ and ‘large reward’ levels (t(41) = 6.112, *P* <0.001) but no difference in RT between the ‘small’ and ‘large’ rewards (t(41) = −1.592, *P* = 0.119). Difference scores (RT ‘small reward’ - RT ‘no reward’; RT ‘large reward’ - RT ‘no reward’; RT ‘large reward’ - RT ‘small reward’) were calculated to examine the group by magnitude interaction. These indicated that the ASD group showed less of a difference in RT between ‘no reward’ and ‘small reward’ (t(40) = −2.337, *P* = 0.025)) and between ‘no reward’ and ‘large reward’ than the control group (t(40) = −2.434, *P* = 0.020) but not between ‘large reward’ and ‘small reward’ (t(40) = −0.809, *P* = 0.424). There was no significant effect of group, group by reward type interaction, magnitude by reward type interaction, or group by reward type by magnitude interaction.

### Accuracy

As discussed above mean accuracy was maintained for all conditions by adjusting the duration of the trigger from trial to trial (see Methods). However, a significant effect of reward magnitude was observed (*F* (1.65, 66.17) = 21.15, *P* <0.0001, that is, greater accuracy for ‘reward’ compared to ‘no reward’), using a mixed model two-by-two-by-three ANOVA (between-subjects factor: group; within subjects factors: reward type and reward magnitude). Pair-wise comparisons indicated a significant increase in accuracy between ‘no reward’ and ‘small reward’ levels (t(41) = 5.76, *P* <0.0001) as well as ‘no reward’ and ‘large reward’ levels (t(41) = 4.52, *P* <0.0001) but no difference between the ‘small’ and ‘large’ rewards (t(41) = −1.00, *P* = 0.323). No other significant effects were observed.

### fMRI results

#### Reward anticipation

Results for the between groups analyses, carried out using a two-by-two mixed model ANOVA (between subjects factor = group; within subjects factor = reward type) are presented in Table 2. There were no significant main effects of group, or group by reward type interactions in *a priori* anatomical regions for reward cues. The interaction of group by reward type in the left anterior cingulate did not survive correction for multiple comparisons with anatomical SVC.

**Table 2:** **Two-by-two mixed model ANOVA (group by reward type) for the contrast correctcue > baseline**

	**Cluster size (voxels)**	**F (Peak)**	**MNI co-ordinates (x,y,z)**	**BA and probability (%)(if available)**
**Main effect of rewardanticipation (MID > SID):**				
**Frontal**				
Right middle frontal gyrus^a^	64	27.63	44, -6, 58	6 (40%)
Left inferior frontal gyrus p. orbitalis^a,b^	242	23.95	−36, 34, -6	47
Left SMA	60	23.41	0, 10, 64	6 (40%)
Left inferior frontal gyrus p. triangularis	33	17.45	−44, 34, 14	45 (50%)
Right inferior frontal gyrus p. orbitalis	31	17.05	42, 26, -10	47
**Temporal**				
Right superior temporal gyrus	43	23.42	56, -26, -2	21
Left middle temporal gyrus	52	19.33	−50, -58, -2	37
Left middle temporal gyrus	12	14.44	−56, -24, -12	20
Left inferior temporal gyrus	14	15.95	−34, -6, -23	20
**Parietal**				
Left postcentral gyrus^a^	90	18.8	−46, -24, 48	2 (50%)
Right precuneus	51	17.99	8, -52, 44	7
Left inferior parietal lobule	29	15.16	−28, -50, 44	SPL 7PC (10%)
Right precentral gyrus	13	15.95	14, -28, 64	4a (50%)
**Occipital**				
Left superior occipital gyrus	33	16.55	−20, -66, 38	SPL 7a (10%)
Right superior occipital gyrus	19	18.31	24, -74, 44	SPL 7P (10%)
Right middle occipital gyrus^a^	2951	33.03	36, -90, 6	17
Left middle occipital gyrus	484	24.21	−32, -90, 12	18 (10%)
**Subcortical**				
Left nucleus accumbens^b^	228	30.25	−10, 2, 0	NA
Right nucleus accumbens^b^	199	27.38	8, 10, 0	NA
Left amygdala	14	15.95	−34, -6, -28	Amyg (LB) 40%
**Cerebellum**				
Right lobule VIIa crus I	49	21.91	32, -76, -30	90%
Right lobule VIIa crus II	10	17.01	8, -86, -38	74%
Left lobule VI	36	21.53	−34, -46, -24	20%
Left VIIa crus 1	32	19.71	−24, -82, -32	99%
**Group by reward typeinteraction:**				
**Frontal**				
Left anterior cingulate	38	16.26	−6, 8, 30	24
**Parietal**				
Left inferior parietal lobule	26	16.42	−36, -44, 46	40; hIP3 (40%)

Results are reported at an uncorrected level of *P* <0.001 (extent threshold 10 voxels).

^a^Regions surviving correction for multiple comparisons (FWE *P* <0.05) at the whole-brain cluster or peak level.

^b^Using anatomical small volume correction in *a priori* regions.

#### Reward feedback

**Figure 3:**
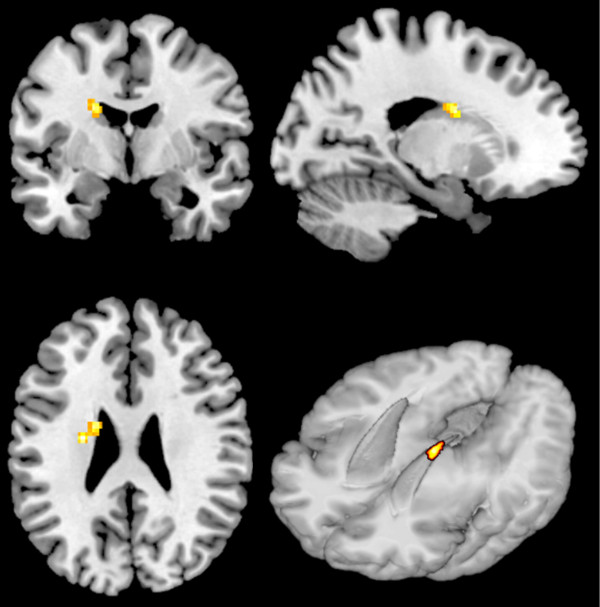
** Group by reward type interaction for reward feedback in the left dorsal caudate.** Results are displayed on a standard brain in MNI space (shown in neurological convention-left is left).

**Figure 4:**
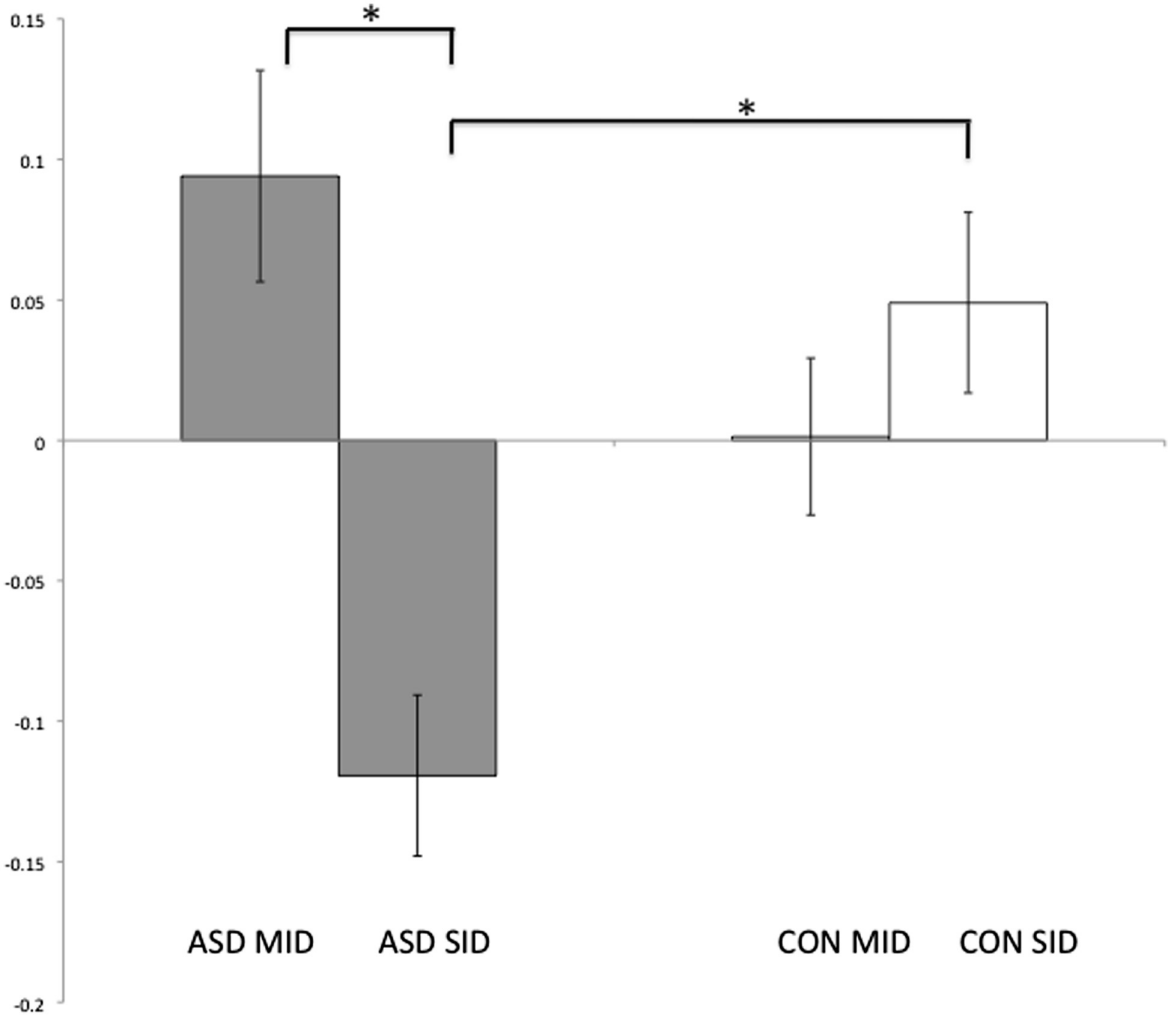
** BOLD response in the left caudate for reward feedback for the SID and MID.** The ASD group is shown in gray and controls in white. The ASD group showed significantly reduced activity compared to controls for the SID. There was no significant group difference for the MID. The ASD group showed significantly decreased activation to social compared to monetary rewards, whereas the controls did not show a significant difference between tasks. Significant within and between group differences are marked with an asterisk and standard error of the mean is displayed.

Results for the two-by-two mixed model ANOVA (group by reward type) are presented in Table 3. There were no main effects of group within reward circuitry but there was a significant interaction in the left dorsal caudate (see Figures 3 and 4) which was corrected for multiple comparisons using an anatomical SVC of the left striatum (MNI co-ordinates: -18, -2, 24; F = 18.62; P_FWE_ <0.05). Independent samples t-tests indicated that the ASD group showed reduced activation, compared to controls, within the same region of left dorsal caudate for the receipt of social rewards (MNI co-ordinates: -16, -2, 24; T = 4.24; P_FWE_ <0.05). Paired samples t-tests also indicated that the ASD group showed a significant difference in activation between the two tasks (reduced activation for SID compared to MID; MNI co-ordinates: -16, 6, 22; T = 4.91; P_FWE_ <0.05) whereas the control group did not. These results suggest that a super-additive interaction within the left DS driven by de-activation to social reward feedback in ASD. This supports our second hypothesis, that reward deficits are specific to social stimuli in ASD, in line with social motivation theory [3]. 

**Table 3:** **Two-by-two mixed model ANOVA [group by reward type] for the contrast correct feedback > baseline**

	**Cluster size (voxels)**	**F (Peak)**	**MNI co-ordinates (x,y,z)**	**BA and/or probability (ifavailable)**
**Main effect of rewardfeedback (MID > SID):**				
**Frontal**				
Right inferior frontal gyrus p. orbitalis	36	17.96	42, 22, -12	45
Right paracentral lobule	35	17.51	10, -28, 64	4a (50%)
Left inferior frontal gyrus p. opercularis	27	15.79	−54, 14, 32	44 (60%)
Left superior medial gyrus	20	13.72	−4, 46, 30	32
Right anterior cingulate	23	15.63	4, 34, 20	24
Right middle cingulate	80	15.18	4, -30, 34	23
Left insula lobe	11	14.41	−32, 24, 4	47
**Temporal**				
Right superior temporal gyrus^a^	81	20.44	66, -24, 6	22; TE 3 (40%)
Right superior temporal gyrus	29	15.78	54, -14, 4	48; TE 1 (70%)
Left superior temporal gyrus	12	14.45	−40, -32, 10	41; TE 1.1 (60%)
Right inferior temporal gyrus	17	16.74	52, -62, -12	37; HOC5 (V5) (10%)
**Occipital**				
Left middle occipital gyrus^a^	834	42.68	−32, -94, 6	18 (20%); hOC3v (V3v) (20%)
Left calcarine gyrus^a^	103	18.49	−16, -74, 8	17 (80%)
Right calcarine gyrus	37	15.2	16, -70, 10	17 (90%)
Right fusiform gyrus^a^	1787	61.63	30, -66, -4	18 (10%)
Right fusiform gyrus	16	18.56	42, -46, -16	37
Left fusiform gyrus^a^	95	59.6	−28, -64, -12	19; hOC4 (V4) (10%)
**Parietal**				
Right postcentral gyrus	34	20.58	62, -14, 30	3b (30%)
Left postcentral gyrus	10	14.4	−46, -24, 44	2 (80%); 3b (60%)
Left inferior parietal lobule	52	15.68	−34, -50, 56	SPL (7PC) (60%)
Left inferior parietal lobule	13	18.91	−54, -30, 38	2; IPC (PFt) (40%)
Left superior parietal lobule	45	17.72	−20, -48, 46	SPL (5 L) (20%)
**Subcortical**				
Right caudate nucleus^b^	17	17.14	14, 8, 20	NA
Right caudate nucleus	12	15.24	16, 12, 0	NA
Left thalamus	15	13.9	−28, -34, 2	Th visual (18%); Temporal (11%)
**Cerebellum**				
Right lobule VIIa crus1	16	14.91	42, -74, -38	72%
**Main effect of group(ASD > CON):**				
**Parietal**				
Left rolandic operculum	11	15.31	−50, 0, 8	43; OP 4 (30%)
**Group by reward typeinteraction:**				
**Parietal**				
Right angular gyrus	21	16.31	32, -64, 46	SPL (7P) (10%)
Left inferior parietal lobule	25	17.21	−32, -52, 44	40; hIP3 (30%)
Right postcentral gyrus	16	14.81	32, -64, 46	3b (60%)
**Temporal**				
Right inferior temporal gyrus	47	20.91	52, -62, -14	37; hOC5 (V5) (10%)
**Subcortical**				
Left caudate nucleus^b^	62	18.62	−18, -2, 24	NA
**Cerebellum**				
Cerebellar vermis lobule VI	21	17.74	−2, -76, -16	71%
Right lobule VIIa crus 1	17	14.69	46, -66, -32	100%

Results are reported at an uncorrected level of *P* <0.001 (extent threshold 10 voxels).

^a^Regions surviving correction for multiple comparisons (FWE *P* <0.05) at the whole-brain cluster or peak level.

^b^Using anatomical small volume correction in *a priori* regions.

#### Correlations between significant BOLD response in the DS and RT

As the DS has previously been implicated in the reinforcement of action [9] and linking rewards to executive functions [12] we investigated whether the BOLD activation (at the co-ordinates described above) was associated with behavioral performance in terms of RT (as accuracy data were held constant). Increased BOLD response for rewards was associated with faster responses for ‘rewards’ compared to ‘no rewards’ in both groups for the SID (r_s_ = 0.367; *P* = 0.017), but not the MID (r_s_ = −0.033; *P* = 0.836), corrected for multiple comparisons (Bonferroni correction, *P* = 0.025).

#### Correlations between significant BOLD response in the DS and clinical variables

There was a significant negative correlation, with higher scores on the ADOS-Stereotyped Behaviors and Restricted Interests scale associated with reduced BOLD signal in the DS region (see co-ordinates above) for social rewards (r_s_ = −0.559; *P* = 0.008) but not monetary rewards (r_s_ = 0.50; *P* = 0.829). The correlation between ADOS-Stereotyped Behaviors and Restricted Interests and BOLD signal in the DS to social rewards did not withstand correction for multiple comparisons (Bonferroni correction, *P* = 0.00357). There were no other significant relationships between ADOS/ADI subscales or the SRS and BOLD signal in the DS.

## Discussion

According to social motivation theory, social deficits in ASD are due to a difficulty in forming reward representations for social stimuli [2,3]. The purpose of this study was to examine whether impaired reward processing in ASD is specific to social rewards or can be generalized to other classes of stimuli and to interpret results in relation to behavioral deficits in ASD. Our results are in line with social motivation theory indicating abnormal processing of social rewards in the left DS during reward receipt in ASD. Specifically, for the feedback condition the ASD group showed reduced activation for social rewards compared to controls in the left DS. The ASD group also showed reduced activation to social rewards compared to monetary rewards in this region (see Figures 3 and 4). Significant results were largely driven by de-activation from the baseline for social rewards in the ASD group. Controls did not show significant activation to social rewards or a significant difference between the two reward types in this region. In terms of the behavioral results, activation to social rewards in the DS was associated with faster responses to social rewards in both groups which is in line with previous studies showing that the DS is important for linking reward processes with executive function [46] and action control [47]. De-activation to social rewards in the DS was associated with higher restricted interests and repetitive behaviors in the ASD group.

### The role of the dorsal striatum in reward processing

The DS is involved in the reinforcement of action [6], playing a fundamental role in goal-directed action through the selection of appropriate goals based on the evaluation of action outcomes [12]. Actor-critic models have informed understanding of striatal function, by positing that the ventral striatum (VS) predicts future rewards (‘the critic’) whereas the DS maintains information about the rewarding outcome of actions (‘the actor’) [48]. In line with this model, it has been found that the VS supports stimulus-reward learning whereas the DS is necessary for stimulus–response-reward learning [47]. The DS plays an important role in updating the reward value of chosen actions to guide subsequent behavior and maximize reward consumption [49,50]. Representations of chosen actions can be used to aid learning or to modulate movements to reflect the value of the action, for example, by modulating RT [50].

In this study, the ASD group had difficulty modulating their RT according to reward level. The ASD group also showed reduced activation compared to controls in the DS for social rewards. Increased BOLD response in the DS to social rewards was associated with faster responses to social rewards in both groups. This suggests that participants with ASD may have difficulty in using social reinforcement to update reward representations and guide subsequent behavior. DS activation to monetary rewards was not associated with faster responses, implying that the region may be more important for social reward processing. Accumulating evidence implicates the DS in processing of complex social rewards such as trust [14], mutual social co-operation [20], receiving positive feedback about one’s personality [17], and altruistic punishment [19,21]. Though the task used in the present study was a simple social reward task, the results further implicate the DS in social reward processing and suggest a deficit in ASD evidenced by deactivation to social rewards in this region.

#### The striatum in ASD

Structural and functional neuroimaging studies have implicated the striatum, particularly the caudate nucleus, as potentially disrupted in ASD. Meta-analyses and cross-sectional MRI studies have reported enlarged caudate volume generally and across age ranges in ASD [51-54]. Striatal white matter abnormalities [55,56] and increased functional connectivity between the dorsal caudate and sensory processing regions [57] have previously been reported. fMRI studies have shown striatal hypo-activation during facial expression imitation and cognitive flexibility tasks [58,59] as well as hyper-activation during sensory-motor tasks [60]. This suggests that the striatum may be more involved in basic sensory-motor tasks in ASD and less in social, communicative, and higher-level cognitive tasks. Striatal abnormalities have typically been associated with restricted interests and repetitive behaviors in ASD [54,61,62], which is in line with the present results whereby striatal de-activation social rewards was associated with increased restricted interests and repetitive behaviors in ASD.

#### Reward processing in ASD: present findings and previous research

Both specific social reward processing deficits [23] and general abnormalities in reward processing have been reported in ASD [24,25]. In the present study, the neuroimaging results indicated social but not monetary reward processing deficits, similar to Scott-Van Zeeland *et al*. [23]. However, behavioral results suggested abnormal processing of monetary rewards as well as social rewards. It is therefore possible that we did not detect subtle between group differences in the neural processing of monetary rewards. Unlike previous studies, we did not detect abnormalities in regions typically associated with incentive motivation (the VS) and the representation of reward value (the OFC and vmPFC). Previous results have been inconsistent (see Introduction) perhaps reflecting the complex nature of reward processing which involves a network of interacting regions [7], or methodological differences between studies. In a number of previous studies, over half of the participants were taking psychoactive medication [5,23,24], which has a known impact on dopamine regulation and by implication reward processing [63] as well as having a potential influence on the BOLD signal [64]. IQ matching and screening for co-morbid psychiatric disorders were not systematically carried out in all studies, introducing other potential confounds. Differences associated with the age and gender of participants may have further contributed to variability between studies as both of these factors are associated with differences in reward processing [13,65]. Here, we sought to address these possible confounds, by only including medication-free male subjects, matching groups on age and IQ, and by co-varying for age and IQ in the fMRI analysis. Subtle differences in task design may further account for some of the discrepancies. For example, for social reward feedback, some studies have contrasted a smiling face with a frowning face [23], whereas other studies [24] including the present study, contrasted a smiling face and a neutral image. Given that the striatum responds to punishment as well as reward [27,66,67], group differences in the VS may have been affected by the negative social feedback and may not have been specific to social reward.

#### Limitations

An important consideration is that the significant group difference during social reward feedback in the DS was largely due to de-activation from the baseline in the ASD group. Though controls showed an increase from the baseline for social rewards this was not significant (see Additional file 1). This may be due to a limitation in the task design and future studies may address this issue by using more robust social reward paradigms (see future directions). A second important limitation is that there was a large age range in the sample. There were no significant age effects in the DS (see Additional file 1), however the large age range invites caution in interpreting negative findings in other regions which undergo pronounced maturational changes [65,68]. Therefore negative results (for example, the lack of group differences in monetary reward processing) may have been due to heterogeneity in the BOLD signal. Additionally, four participants who had ADHD/ADD diagnoses secondary to an ASD diagnosis were included in the study. As ADHD is associated with aberrant reward processing [69,70], analysis was repeated without these participants. Results remained significant at an uncorrected level suggesting that group differences were not attributable to the presence of these subjects but that their inclusion was necessary to have sufficient statistical power to correct for multiple comparisons.

Correlations with behavioral impairments, as measured by the SRS, ADOS, and ADI were exploratory. Caution is warranted in interpreting the correlation between ADOS-Stereotyped Behaviors and Restricted Interests and the BOLD signal in the DS as it did not survive correction for multiple comparisons. Numerous studies have previously used the ADOS and ADI to measure behavioral impairments in ASD [55,71,72] but findings are limited by the fact that these are diagnostic scales with ordinal values. As in a previous study we combined the child and adult versions of the SRS [73]. Though there are no published data on the clinical validity of the adult SRS, a recent study has supported its genetic validity showing that it measures a quantitative, heritable trait [73].

#### Future directions

These results open several avenues for future research. Reward processing undergoes maturational changes between adolescence and adulthood in typical development [65,68], therefore examining developmental factors will be important in future studies of reward in ASD. Gender differences have also been reported in reward processing [13], therefore future studies could investigate whether the same gender differences apply to women with ASD. We did not find a significant correlation between the BOLD signal during social reward processing and social impairment in ASD. One study reported a correlation between BOLD signal in the striatum and social functioning in controls but this relationship was not observed in ASD [23]. Therefore further study is needed to evaluate whether deficits in social reward processing are associated with social impairments in ASD. Social reward paradigms with dynamic stimuli [74] and multi-modal information (verbal and auditory) [18] may be more rewarding for participants and could be useful in future studies of social reward in ASD. Finally, more complex social decision-making tasks may provide a link between reward processing and theory of mind deficits in ASD [75].

## Conclusions

Our data indicate, in line with social motivation theory, that ASD is characterized by abnormal striatal responses to social rewards, that the more de-activation in this region to social rewards the greater number of restricted interests and repetitive behaviors in ASD, and that increased activation in this region is correlated with faster responses to social rewards in both ASD and controls.

## Abbreviations

ACC: Anterior cingulate cortex; ASD: Autism spectrum disorder; BOLD: Blood-oxygen-level-dependent; DS: Dorsal striatum; fMRI: Functional magnetic resonance imaging; OFC: Orbitofrontal cortex; RT: Reaction time; vmPFC: Ventromedial prefrontal cortex; VS: Ventral striatum.

## Competing interests

Authors have no competing interests to declare.

## Authors’ contributions

SD conceived of the study, recruited participants, carried out assessments and data collection, performed the statistical analyses, and drafted the manuscript. JHB contributed to the design of fMRI paradigms and advised on data analyses. LG participated in the design of the study and carried out consensus clinical diagnosis. JMcG, JF, and SB contributed to data collection, AJF contributed to the MRI acquisition. All authors helped to draft the manuscript and read and approved the final version.

## Supplementary Material

Additional file 1 Supplementary materials. Click here for file
