# Supplementary material for: Social and monetary reward processing in autism spectrum disorders
Source: Mol Autism. 2012 Sep 26;3:7. doi: 10.1186/2040-2392-3-7 (PMC3499449; doi:10.1186/2040-2392-3-7)
Supplement: Additional file 1 — Supplementary materials. [file 2040-2392-3-7-S1.docx]

**Selection of Face for Social Reward Task.**

20 Caucasian males (mean age = 29.3; SD = 4.71) were shown the 8 female Caucasian faces in the NimStim set of facial expressions. Each model was presented with all three ‘Happy’ expressions (Happy-Closed Mouth; Happy-Open Mouth; Happy-Exuberant). The participants were asked to choose the model with the most pleasant face. When evaluating how pleasant the faces were, they were asked to consider both how attractive and how pleasant they found the picture based on their facial expression. They were then and were asked to rate each one individually in terms of how pleasant they found the faces on a scale of 1-10 where 1 represented ‘very unpleasant ‘ and 10 represented ‘ very pleasant’.

Model 01 was chosen as being most pleasant by 55% of raters. Total ‘pleasantness’ scores for each face was calculated from the individual pleasantness ratings of each of the three happy faces for each actor (one to eight). Using Friedman’s ANOVA, there was a significant difference in the ‘pleasantness’ ratings for the different faces (χ^2^ (7) = 36.08; p<.05). Model 01 was rated as being most pleasant overall. Using a Wilcoxen signed rank test, a significant difference was observed between ratings of face number 1 and the next most pleasant face , face number 6 (which received a ‘most pleasant rating from 15% of participants) (T=38.5; p<.05; r = -0.325). Based on these results, model number one was chosen as having the most pleasant face.

Selection of most pleasant facial expression:

Friedman’s one way ANOVA was used to examine differences in pleasantness ratings for all of the Caucasian faces at each of the three levels of Happiness. A significant difference was found between the pleasantness ratings for three levels of Happy faces (χ^2^ (2) = 15.688; p<.05). Wilcoxen signed ranks test showed that Happy-Open faces were rated as more pleasant than Happy-Closed and Happy Exuberant faces (T = 8.5; r = -.45 ; p<.0167; T = 9.5 ; r = -.44; p<.0167 respectively) and that there was no significant difference in ratings of Happy-closed and Happy-Exuberant faces (T = 57 ; r = -.197; p<.0167; T = ; r = ; p<.0167).

Friedman’s one way ANOVA was then used to investigate whether there were significant differences in pleasantness ratings for model 01 at each of the three levels of Happy. A significant difference was found between the pleasantness ratings for three levels of Happy faces (χ^2^ (2) = 13.368; p<.05). Wilcoxen signed ranks test showed that the Happy-Open face was rated as more pleasant than Happy-Closed and Happy Exuberant face (T = 4.5; r = -.46; p<.0167; T = 25; r = -.39; p<.0167 respectively) and that there was no significant difference in ratings of Happy-closed and Happy-Exuberant faces (T = 61; r = -.14 ; p<.0167).

Summary

Face number one was rated as the most pleasant of the eight female Caucasian faces. Happy-open faces were rated as significantly more pleasant than Happy-closed and Happy-exuberant faces. Therefore, model 01 was selected to present feedback in the SID task with the Happy-Closed face used as a small reward and the Happy-Open face used as a larger social reward.

**Within group effects for Social Reward in the Left Dorsal Striatum (DS)**

The ASD group showed significantly reduced activation for social reward feedback from the baseline in the left DS (F= 3.42; extent threshold: >10 voxels; p<.001 uncorrected). Controls did not show a significant difference between the social reward feedback and the baseline (see figure below).

(Insert supplemental figure here)

**Supplemental Figure.** BOLD response to social reward feedback at peak co-ordinates for the group by reward type interaction (MNI =-18 -2 24) as indicated by the cross hairs. The ASD group is shown in red and controls are shown in blue. The ASD group showed significant deactivation compared to baseline for social rewards whereas controls did not show a significant difference from the baseline.

**Age effects in the left DS**

Age was not associated with percent signal change in the left DS for either the ASD (MID: p=.090; SID p=.132) or control groups (MID: p=.653; SID p=.527). Using a 3-way ANOVA (factors: age, group, reward type) there were no significant main effects of age (p=.671) or interaction effects between age and group (p=.952) or age and reward type (p=.084) on percent signal change in the left DS
